# Overview of management of acute renal failure and its evaluation; a case analysis

**Published:** 2015-01-01

**Authors:** Chaudhary Muhammad Junaid Nazar, Faisal Bashir, Saba Izhar, John Anderson

**Affiliations:** ^1^Department of Endocrinology, University of Buckingham, Royal Gwent Hospital, NHS Trust, Wales, UK; ^2^Department of ENT, New City Teaching Hospital, Mohetarma Benazir Bhutto Shaheed Medical College, Mirpur Azad Kashmir, Pakistan; ^3^Department of Internal Medicine, Allma Iqbal Memorial Teaching Hospital Sialkot, Punjab, Pakistan; ^4^Division of Medical Education, Postgraduate Medicine Brighton and Sussex Medical School University of Brighton, Brighton, UK

**Keywords:** Acute tubular necrosis, Glomerular filtration rate, Hemodialysis

## Abstract

The annual incidence is about 150 per million in the UK, but this figure is six times greater in the >80 years old group. Prerenal azotemia is considered as the most serious reason in community or hospital acquired acute renal failure (ARF). A 67-year-old middle age male was admitted to the hospital with a chief complaint of generalized weakness, volume depletion and dysuria. He has treated with metronidazole for diarrhoea caused by Clostridium difficile considered as the precipitating factor for the ARF. The patient has severe osteoarthritis and takes high dose non-steroidal anti-inflammatory drugs from the last two years. He also complains for obstructive sleep apnea (OSA) and obesity. He has controlled hypertension was on lisinopril to control blood pressure. ARF is quite common, occurring in 80 million populations. Urinary obstruction should be excluded (a cause in around 5-10 of cases) because this is readily reversible if it is diagnosed early. A renal US will be sufficient to identify obstruction in 95 of cases. Most cases of ARF are expected to pre renal failure/acute tubular necrosis (ATN) 70-80%. Risk factor for development for at ATN are old age, drugs (non-steroidal anti-inflammatory drugs, gentamicin), sepsis, and chronic kidney disease and must be considered.

**Implication for health policy/practice/research/medical education:**

Acute renal failure (ARF) is defined as the rapid decline in kidney function as manifested by a reduction in glomerular filtration rate. It is a more frequent problem observed in all hospital admission. The incidence of ARF increases with age; the annual incidence is about 150 per million in the UK, but this figure is six times greater in the >80 years old group. The overall mortality associated with the ARF is higher with hospital acquired ARF. Therefore, better understanding and early detection can help in better prognosis.

## Introduction

Acute renal failure (ARF) is defined as the rapid decline in kidney function as manifested by a reduction in glomerular filtration rate (GFR). It is a more frequent problem observed in all hospital admission. The incidence of ARF increases with age; the annual incidence is about 150 per million in the UK, but this figure is six times greater in the >80 years old group (1). Pre renal azotemia is considered as the most serious reason in community or hospital acquired ARF. The overall mortality associated with the ARF is higher with hospital acquired ARF (2). The case study of ARF is discussed to develop a better understanding by studying the current research project. The discussion will be more focused on the pathophysiology and the new ways of treatment used in current practice.

## Case presentation

A 67-year-old middle age male, was admitted to the hospital with a chief complaint of generalized weakness, volume depletion and dysuria. He has treated with metronidazole for diarrhea caused by *clostridium difficile* considered as the precipitating factor for the ARF. The patient has severe osteoarthritis and takes high dose non-steroidal anti-inflammatory drugs (NSAIDs) from the last 2 years. He also complains for obstructive sleep apnea (OSA) and obesity. He was using lisinopril to control his hypertension. He has five siblings with no significant medical history.

On physically examination, he was clinically volume depleted with a pulse rate of 100 beats per minute. He was dehydrated with dry mucous membranes and reduced skin turgor. His body temperature was 37.8 °C, BP; 105/55 mmHg lying, and 90/50 mmHg sitting. Jugular venous pluse not visible. He was in ARF with serum urea and creatinine of 79 mg/dl and 2.4 mg/dl respectively. He has hypokalemic alkalosis with a potassium level of the 1.4 mEq/l (3.5-5.0 mEq/l) and a bicarbonate level of the 41.1 mEq/l (22-28 mEq/l) He was also hyponatremic, sodium level of the 125 mEq/l (136-145 mEq/l) but his serum calcium level was within normal range.

Renal ultrasound showed the right kidney measuring 12.4 cm and left kidney measuring 12.1 cm, with no signs of the shadowing calculus or hydronephrosis. However, it showed the presence of the simple bilateral cyst. Urine dipstick results showed protein of +++ and no blood. A 24 h urine sample showed nephrotic range proteinuria with proteins of 6.48 g/24 h, but serum albumin level was normal at 3.6 g/dl. His hemoglobin was 13.3 g/dl. WBC=11.9×103/µ and platelet count was normal.

### Diagnosis

The history may point out to the cause of ARF (e.g. drugs, skin rash); assessment of the hemodynamic is crucial, and proper fluid resuscitation should be given. There are different sign and symptom including hypotension, hypovolemia and his dehydration state that give indication to the diagnosis. Fractional excretion of sodium (FENa) was calculated and its value was 0.77%. It is generally less than 1% in patients with acute glomerulonephritis, hepatorenal syndrome, and states of prerenal azotemia such as congestive heart failure or dehydration. This value gives confirmation of prerenal failure.

Renal ultrasound ruled-out urinary obstruction. The diagnostic specificity of FENa in differentiating prerenal azotemia from the interarenal cause of the ARF may also be influenced by the fact that the patients may actually be progressing from the prerenal azotemia state to established ARF. The drug history of the patients can also affect the values of FENa. Despite of these many limitations, FENa when it is considered as an important tool in the context of the other clinical scenario (3).

There is considerable interest in the potential utility of the different blood and urinary biomarker, which can be important for the diagnosis of ARF. Biochemically, serum creatinine and blood urea nitrogen (BUN) concentration are important diagnostic tool in the detection of ARF. An abrupt increase in serum creatinine concentration usually reflects a decrease in GFR and signals the occurrence of ARF. These two diagnostic tools are affected by the condition and have some limitations e.g. gender, muscle mass and the drugs so they are not fully reliable (4). Furthermore, serum creatinine concentration does not accurately influence GFR in the unsuspecting state of ARF. On the other hand, increased urea validity, gastrointestinal bleeding, protein intake, catabolic states, protein malnutrition and cirrhosis hit BUN values, which can lead to wrong diagnosis (4). Cystatin-C and alpha 1-microglobulin, are diverse tools on which research work is going on to find more reliable and efficient tool to diagnosis ARF early. Studies have shown cystatin-C to be an early and reliable marker of acute kidney injury (AKI) in patients in the ICU but it is not the validated as GFR indicator in ARF as compared to serum creatinine and still its requires further studies (5). Similarly, N-acetylglucosaminidase, KMI-1, neutrophil gelatinous associated lipocalin (NGAL) are under trial to find the fast and timely detection of ARF (5).

### Urinary examination

Assessment of urine biochemistry is essential and inexpensive tool in the evaluation of AKI. The factors which need to be considered important are elaborated in Table 1.

**Table 1:** Assessment of urine biochemistry

**Urinary findings**	**ATN**	**Prerenal uremia**
Urine sodium	40 mEq/l	20 mEq/l
Urine/Plasma osmolality	1.1/ 1	1.5/1
FENa	1%	1%
Urine/Plasma urea	7/1	10/1
Urine volume	Oliguric	1.5

FENa‏= Fractional sodium excretion; ATN= Acute tubular necrosis

### Autoantibody profile

Antinuclear factor (ANF), anti-neutrophil cytoplasmic autoantibody (ANCA), anti-GBM, complement and urinary electrophoresis should be performed unless the cause of AKI is obvious, e.g. post myocardial infarction or renal obstruction.

### Percutaneous renal biopsy

Renal biopsy is important diagnostic tool for those patients in whom prerenal and post renal ARF have been excluded. It helps further to rule out the cause of the intrinsic renal failure rests unclear e.g. vasculitis, glomerulonephritis, and interstitial nephritis.

### Clinical follow up

On volume resuscitation, the patients became profoundly polyuric, with daily urine output of 7 to 10 liter. He needed large amounts of potassium and magnesium supplements, initially intravenous and later oral. The health of patient improved with correction of potassium and polyuria. Kidney function improved progressively, with creatinine level decreasing to 1.5 mg/dl during the course of 2 weeks. The proteinuria dramatically improved with the optimization of fluid level and electrolyte along with 24 h urine protein decreased to 0.73 g/24 h. The patient had good urine output with intravenous fistula and was in a positive fluid balance. However his mobility is much decreased due to malnutrition and weakness. The patients progressed a lot after treatment of six months and remain well with slight proteinuria but mild kidney function. His kidney serum creatinine was 1.6 mg/dl. His stool was *clostridium difficile* toxin positive. His diarrhea gradually improves over the next few days with metronidazole. Plasma renin and aldosterone concentration ambulant was grossly increased. On discussion with the multidisciplinary team he is unlikely to improve sufficiently to go back to his own house but require sheltered accommodation or even a residential home.

## Discussion

The first dispute to resolve is whether the renal failure is likely to be acute or chronic. Patient’s kidneys are on the small side and his creatinine was elevated on admission giving a GFR indicative of 20 ml/min/1.73 m. Although there may be chances of chronic kidney disease (CKD) in this case, the current history is acute reduction of urine output in the last 12 h and a precipitous increase in creatinine, in keeping with ARF. Therefore, this is likely to be an acute on chronic renal failure. There are many causes of ARF classified listed in Table 2.

**Table 2:** Causes of acute renal failure

Reduced circulating volume (60%)	1. Blood loss, excess gastrointestinal losses, burns, low cardiac output, toxic or ischemic myocardial depression 2. Drug induced renal profusion angiotensin converting enzyme inhibitors, non- steroidal anti-inflammatory drugs) 3. Large vessels e.g. renovasculature disease), small vessel occlusion: disseminated intra-vasculature coagulation; hemolytic uremic syndrome (HUS)
Toxic ATN (5%)	Rhabdomyolysis with urinary myoglobin, Radio contrast nephropathy
Structure abnormality of renal vasculature (15%)	Large vessels e.g. renovascular diseases
Acute glomerulonephritis and vasculitis (15%)	ANCA –positive vacuities, Goodpasture syndrome
Interstitial nephritis (5%)	
Myeloma/tubular cast nephropathy (5%)	

ANCA= anti-neutrophil cytoplasmic autoantibody; ATN= acute tubular necrosis

But in this case the renal ultrasound shows no sign of the hydronephrosis, which might indicate obstruction. If obstruction is excluded then the most likely case is pre-renal failure. The prerenal failure will continue to the acute tubular necrosis (ATN) if left untreated. ATN occurs if there is nonstop hypovolemia, hypotension and exposure to nephrotoxic drugs or sepsis.

### Pathophysiology of ATN

After an ischemic injury abuse there is forceful arterial vasoconstriction, facilitated by the release by the vasoconstriction (particularly endothelium and by the loss of intrinsic vasodilators (nitric oxide and prostaglandin I2 (PGI); this contributes to the loss of GFR and the restructuring of blood flow within the kidney. Hypoxic injury to the power–consuming cells of the proximal tubule and thick ascending limb of loop of henle occurs, then calcium and oxygen free radical mediated cell necrosis results in cell shedding from the tubular basement membrane, with the formation of the cast that block urine flow. Patients with suspected acute tubular necrosis are not routinely biopsied unless with further kidney pathology is suspected. A number of clinical features in this case are likely causes of ARF including his obvious dehydration and hypotension. Especially the patient was also using NSAIDs for long duration, which can be considered as an imperative factor in the causation of the AKI. NSAIDs (NSAIDs) are common cause of AKI used by the people either prescribed or bought over the counter (6). However, there is little evidence that NSAIDs own a role in the impairment of renal function of normal healthy person. However, in specific clinical setting such as atherosclerotic cardiovascular diseases in old age people, diuretic use, pre-existing chronic renal failure and NSAID using, AKI could be induced. Furthermore, study regarding NSAIDs, showed, this kind of AKI is reversible within 3-7 days when this drug is discontinued. Less frequently NSAIDs can cause acute tubular necrosis or even, more rarely papillary necrosis (7). There are many other drugs, which can be nephrotoxic and can play an important role in the pathogenesis of AKI such as angiotensin-converting enzyme inhibitors (ACEIs) and angiotensin receptor blockers (ARBs) if used in combination can increased risk for post-operative renal dysfunction, possibly as a result of the intraoperative hypotensive episodes (7). Similarly, other drugs such as gentamicin or amphotericin can be nephrotoxic in order (7).

### Management of acute renal failure

The mainstay of the management involves the increase of the fluid balance hemodynamic stabilization with the optimization of the cardiac output and blood pressure is considered as the most effective steps in the treatment of the acute renal failure. Initial fluid management is important intervention for the ARF patient and to prevent further injury e.g. hypotension and hypovolemia. Assessment of the volume level is challenging, especially patients in intensive care units (1). There are no specific guidelines for the increasing hemodynamic and fluid level for the renal function protection, but predetermine of data from the clinical setting associated with ARF can be informative. However to improve the assessment of volume status, international guidelines for the management of the sepsis from the surviving sepsis strategy recommended invasive monitoring with the measurement of central venous pressure and venous oxygen saturation (superior vena cava or mixed) based on the first goal-directed treatment approach can be helpful (8). However, there is debate about the optimal fluid to use for resuscitation in critically ill patients. The recent saline versus albumin fluid assessment safe trial of 6,997 patients found that fluid resuscitation with saline or albumin resulted in similar relative risk for death in critically ill patients (9) and avoidance of either hypovolemic or fluid overload. Blood pressure should be controlled, hemoglobin maintained above 9 g/dl and sepsis should be quickly and vigorously treated. In conclusion, a flexible fluid method as part of early goal directed therapy appears to be beneficial during the first 6 h. However, the potential risk of the fluid accumulation must be considered in the setting of ARF (10). Renal dose dopamine loop diuretics are often used in the acute tubular necrosis, although there is no evidence that they it can change the outcome of ARF in humans. Renal dose dopamine 0.5 to 3 mcg/kg/min given as specified vasodilator to increase blood flow and to avoid AKI increases urine output but does not disturb AKI outcome or mortality (11). Some cases of the ARF can be managed without dialysis, with the adoption of alert fluid balance and dietary restriction.

Key to ARF management is the devotion demanding nutritional support of the sicker patients, and the use of the continuous renal replacement (e.g. CVVH: continuous veno-venous hemofiltration), which are less likely to produce hemodynamic instability.

Other more specific treatments in ARF depend upon the causative form and include the following:

1. Specific immunosuppressive therapy and sometimes plasma exchange may be appropriate for some condition like goodpasture syndrome, ANCA positive vasculitis.
2. Obstruction: Bladder catheterization if there is urine outflow congestion, nephrostomy drainage for renal obstruction.
3. Other: e.g. steroids in acute interstitial nephritis (AIN), plasma exchange in hemolytic uremic syndrome (HUS) and Thrombotic thrombocytopenic purpura (TTP), chemotherapy in myeloma.

There are different drugs, which are used, in ARF listed in Table 3 along with level of evidence but still a lot of additional research going on to proof their effectiveness.

**Table 3:** Summary of drugs used in treatment of acute kidney injury

**Drugs**	**Level of evidence**	**Result**
Loop diuretics	RCTs and meta-analysis	No effect
Atrial natriuretic peptide	RCTs	Possible beneficial on survival and kidney function
Dopamine	RCTs	No effect on mortality or kidney function
Norepinephrine	Prospective observational studies	Possible beneficial effect on kidney function
Fenoldopam	1. RCTs 2. One meta-analysis	1. No effect on mortality or kidney function 2. Beneficial effect on mortality and need for dialysis.
Insulin	Meta-analysis	Controversial effects
Mesenchymal stem cells	Animal models	Beneficial effect on kidney function
Erythropoietin	Animal models	Beneficial effect on kidney function

Randomized controlled trials

### Emerging agents

There are numerous randomized trials (Table 4) going on of different drugs such as calcium channel blockers, adenosine antagonist, multipotent stem cells and erythropoietin to check out their efficiency in the treatment of acute renal failure. Calcium channel blockers have shown some effects to alter the afferent arteriolar vasoconstriction induced by a variety of stimuli and also natriuretic result (9). In large multi-central randomized control trial to investigate the effectiveness of isradipine on renal function, incidence and severity of delayed graft rejection was done. It did not work and found no benefits (3). Similarly, small clinical studies assessing the role of the theophylline, an adenosine antagonist, in the prevention of the contrast nephropathy have shown some different effect (12). There is on going research project looking at the effects of erythropoietin (EPO) or placebo on the prevention of AKI in patients under going heart surgery or kidney transplantation. In the intensive care setting the study failed to show therapeutic renoprotective benefits of EPO however there were obvious flaws in the study the patients do not receive the medication on time and secondly extreme of EPO was used against the AKI patients but it did not alter the outcome (13).

**Table 4:** Summary of drugs used in prevention of acute kidney injury

**Drugs**	**Level Of evidence**	**Results**
Dopamine	RCT s	No effect on kidney function
Fenoldopamine	1.Small RCT 2.One meta analysis	1. No effect on kidney function 2. Beneficial effects on kidney
Loop of diuretics	RCTs and meta analysis	No effect on kidneys function
N-Acetlycestine	RCTs and meta analysis	Variable beneficial effects on kidney function
Statins	Animal mode	Beneficial effect on kidney
Calcium channel blocker	RCT in peri-transplant period	No effect on kidney function
Adenosine antagonist	RCTs	Controversial effect on kidney function
Multipotent stem cells	Animal models	Beneficial effects on kidney function
Erythropoietin	Animal models	Beneficial effects on Kidney
Small interfering RNA targeting p53	Animal models	Beneficial effects on kidney function

Randomized controlled trials

### Prognosis and outcome of dialysis in ARF

The overall survival for patients with ARF remains relatively limited, 55-60% of the patients require dialysis treatment survive, but the numbers partly reflects the very poor outcome of the patients with who have ATN as a component of multiple organ failure (MOF) who are managed on the ICU. The registry data specified the lower risk of peritoneal dialysis as compared to hemodialysis during the first year of treatment (14). For example, only 10-20% for those with three or four organs failure will survive, yet 90 patients who have ARF in isolation survive. The survival speed fluctuates depending upon essential cause of end stage renal disease, age, and associated comorbidities e.g. cardiovascular diseases, diabetes and hypertension. One study performed by the Medicare in US shown HD is associated with increase chances of death among diabetic patients as compared with those patients without any co-morbidities (15). Indications for urgent dialysis in ARF was summarized in Table 5.

**Table 5:** Indications for urgent dialysis in ARF

Severe uremia	Uremic encephalopathy
Hyperkalemia	Potassium >6.5 mEq/l or lies, if ECG changes apparent
Severe acidosis	
Uremic pericarditis	
Pulmonary edema	

The prognosis for the recovery of renal function varies according to the causative condition; renal recovery occurs <50% of cases with autoimmune vacuities. In survivor for ATN, renal function will return to the normal range in 60%, whereas 30% will be left with CKD and 10% will be dialysis–dependent.

## Conclusion

ARF is quite common, occurring in 80 million populations. Urinary obstruction should be excluded (a cause in around 5-10 of cases) because this is readily reversible if it is diagnosed early. A renal US will be sufficient to identify obstruction in 95 of cases. Most cases of ARF are expected to pre-renal failure/ATN 70-80%. Risk factor for development for at ATN are old age, drugs (NSAIDs, ACEIs and gentamicin), sepsis, CKD. If obstruction has been excluded and there is nothing suggests a more unusual, renal cause of ARF, then ATN is the most likely diagnosis and patient should be treated with intravenous fluids to restore intravascular volume. The underlying cause of hypotension should be treated and any nephrotoxins must be removed. If blood pressure remains low following an adequate filling then the patients may require inotropic support, which will require an ITU bed. If intravenous rehydration restore intravascular volume and blood pressure but there is no improvement in oliguria, this is likely to be established acute tubular necrosis and the patient may require a period of renal support (hemodialysis or filtration) whilst tubular cells regenerate. This usually takes days to weeks but can take months. ARF in the elderly has a significant mortality, particularly if the patients require renal replacement therapy.

## Authors’ contributions

CMJN and FB completed the article. SA and JA done critical appraisal.

## Ethical considerations

Ethical issues (including plagiarism, misconduct, data fabrication, falsification, double publication or submission, redundancy) have been completely observed by the authors.

## Conflicts of interests

There were no points of conflicts.

## Funding/Support

None.
